# The Ecological Drivers of the Mosaic Structure of Bryophyte and Vascular Plant Cover in the Rich Fens of Lithuania

**DOI:** 10.3390/plants14172662

**Published:** 2025-08-26

**Authors:** Monika Kalvaitienė, Ilona Jukonienė

**Affiliations:** State Scientific Research Institute Nature Research Centre, Akademijos St. 2, LT-08412 Vilnius, Lithuania; ilona.jukoniene@gamtc.lt

**Keywords:** *Calliergonella cuspidata*, *Carex lepidocarpa*, *C. rostrata*, *Hamatocaulis vernicosus*, hollows, hummocks, *Scorpidium cossonii*, species pools, topography

## Abstract

The composition and structure of vegetation have been recognised as the main determinants of habitat quality, which influences biodiversity. The presented research focuses on the mosaic structure of Lithuanian rich fens and their relationship to ecological conditions. This study was conducted across 98 study plots amongst 15 fens distributed throughout Lithuania. This research included the cover and abundance of vascular plants and bryophytes, water parameters (conductivity, pH, and concentrations of Ca^2+^, Fe^3+^, K^+^, Mg^2+^, NH_4_^+^, NO_3_^−^, and PO_4_^3−^), topography type, and the cover of hummocks. Vegetation studies resulted in the distinction of two clusters containing ten bryophyte groups and two clusters containing eleven vascular plants groups. The main diagnostic species for bryophyte clusters were *Scorpidium cossonii* and *Calliergonella cuspidata*, and those for the vascular plant clusters were *Carex lepidocarpa* and *Carex rostrata*. The mosaic distribution of vegetation observed in both the bryophyte and vascular plant layers is primarily shaped by local hydrological regimes, microtopographical variation, and the amount of iron present. The habitats of bryophyte groups, as compared to those of vascular plants, were determined by narrower ecological conditions. This study emphasised the specificity of Lithuanian fens, which are located at the junction of the boreal and continental biogeographical regions.

## 1. Introduction

Preserving and restoring the rich biodiversity of Europe is one of the top priorities of the EU [1]. A key component of this effort is the conservation of habitats, which plays a vital role in the protection and sustainable management of natural environments. Ensuring habitat quality is essential for the survival and population growth of endangered species, as well as for maintaining ecosystem services [2,3,4,5]. The composition, structure, and coverage of vegetation have been recognised as the main determinants of habitat quality, directly influencing biodiversity [6].

Rich fens, like all mires, play an important role in providing hydrological, water purification, and carbon sequestration services [7]. Moreover, they harbour the highest biodiversity among wetlands [7,8].

Rich fen occurrence is closely linked to calcareous groundwater sources and stable hydrological conditions [9]. The most suitable regions for the formation and development of rich fens in Europe are found in the northern and central European regions, particularly in the Baltic States, Scandinavia, and parts of Central and Eastern Europe [10]. However, throughout much of Europe, rich fens have undergone a significant decline. For many decades, considerable effort has been put into converting these unused lands into agricultural land [7]. Although direct destruction has become less common recently, fens remain among Europe’s most threatened ecosystems due to various human-induced and interacting factors, including acidification [9,11,12,13,14,15], eutrophication [12,16,17,18,19,20,21], groundwater pollution [22], hydrological disturbance [23,24], the spread of invasive species [25,26,27], climate change [28,29], or the absence of traditional activities (management) necessary for their survival [14,30,31,32]. Many remaining sites are now fragmented and considered conservation priorities at both the national and EU levels. As priority habitats for nature conservation (7140 transition mires and quaking bogs, 7230 alkaline fens, 7160 fennoscandian mineral-rich springs and spring fens, and 7210 calcareous fens with *Cladium mariscus* and species of *Caricion davallianae*), they are included in EU Habitats Directive Annex 1 [33]. The species *Hamatocaulis vernicosus*, *Saxifraga hirculus*, *Liparis loeseli*, and *Meesia longiseta*, which are adapted to the unique rich fen conditions, are listed in EU Habitat Directive Annex II [34].

Although numerous studies have addressed various aspects of rich fens, including their vegetation [35,36,37,38,39,40], ecology [19,41,42,43], and restoration [7,14,23], there is still considerable scope for research to gain a deeper understanding of the ecology of rich fen vegetation and the patterns of its formation and distribution. In particular, there is a lack of comprehensive knowledge not only from broad regional studies but also from detailed local investigations, all of which form the basis for broader studies and generalisations. They are essential for developing more effective conservation and management strategies tailored to specific local conditions.

The mosaic nature of fens is another frequently underestimated feature in the studies of these ecosystems. Rich fens are structurally diverse, harbouring ecologically unique species assemblages (pools). Even within individual areas of fens, variations in ecological conditions give rise to mosaic vegetation expressed as a composition of different plant species or their predominance. This is typically associated with heterogeneity in hydrology, buffering capacity, nutrient supply, and peat accumulation or successional changes in plant communities [7]. Research into the compositions, sizes, boundaries, and spatial relationships of vegetation patches is key to understanding how specific ecosystems function and how they can be conserved [44]. The effective management and restoration of habitats are not sufficient without considering all landscape mosaic structures [44,45,46,47]. Furthermore, introducing desired ecosystem patches to degraded landscapes to promote biodiversity recovery seems to be a promising active restoration strategy [47].

Our previous studies on habitats of the EU Habitat Directive Annex II species *Hamatocaulis vernicosus* have revealed [48] that rich fens in Lithuania stand out from other European regions. Occurring at the junction of boreal and continental regions and providing habitats for the EU Habitat Directive Annex II species *Hamatocaulis vernicosus*, *Liparis loeselii*, and *Saxifraga hirculus*, Lithuanian fens are of high conservation value in the broader European context. Nevertheless, the fens of this region are still under investigation. Among European rich fen studies, most of which are from Central Europe [19,31,37,38], this region is very poorly represented. The exception is research conducted in the northeastern part of Poland [49,50]. Some research on rich fens is carried out in Latvia [51].

The presented research focuses on the mosaic structure of Lithuanian rich fens and their relationship to ecological conditions in a regional and local context. The main questions we aimed to answer were as follows: (i) What are the main species pools of the vascular plants and bryophytes in the mosaic vegetation structure of Lithuanian rich fens? (ii) What are the diagnostic species that distinguish individual pools of bryophytes and vascular plants? (iii) What are the environmental characteristics related to each species assemblage? (iv) Do the same ecological factors determine the species composition in bryophyte and vascular plant pools? (v) What are the characteristics of Lithuanian rich fens in terms of bryophyte and vascular plant composition in the context of other European regions?

## 2. Materials and Methods

### 2.1. Study Site

Lithuania is situated in the transition zone between oceanic and continental climates in Northeastern Europe. This geographical location results in moderate seasonal variations and climatic conditions favourable for the development of peatlands [52]. The country’s average annual precipitation is around 695 mm, and the mean annual air temperature is approximately 7.4 °C. However, recent decades have shown a warming trend, which could affect wetland hydrology and carbon dynamics.

Lithuania is characterised by a relatively flat terrain shaped during the last glacial period, resulting in numerous depressions and poor drainage areas. As a result, peatlands cover approximately 7.4% of the country’s territory, encompassing a variety of mire types, including fens (5.8%), transitional mires (0.7%), and raised bogs (0.9%) [53]. Fens are particularly widespread in low-lying river valleys and glacial depressions (the highest concentrations found in the southeastern and western parts of the country), where groundwater discharge and mineral-rich waters support diverse and specific vegetation communities [54].

### 2.2. Vegetation and Environmental Data Sampling

**Figure 1:**
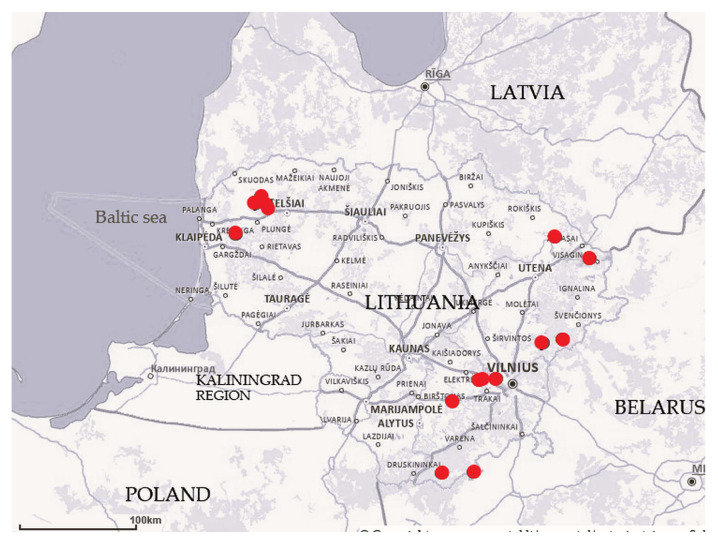
Map of studied fens (n = 15) in Lithuania (base map: https://www.geoportal.lt/map/ (accessed on 25 July 2025)).

This study was carried out during the summer months (June to August) from 2014 to 2019 in 15 fens across various regions of Lithuania (Figure 1). As our aim was to cover vegetation patches that differed in terms of bryophyte and vascular plant composition, we employed a preferential sampling procedure. The study plots were chosen based on the mosaic vegetation structure of areas that were distinct in terms of the following: 1) the dominant species of vascular plants or bryophytes, 2) the shrub cover, and 3) the microtopography. The number of study plots (4 m × 4 m) ranged from two to 15, depending on the size and heterogeneity of the fens according to the above-discussed criteria. A small number of study plots (two to three) were usually established to supplement the already well-described and well-represented vegetation in nearby fens. A total of 98 plots were surveyed (Table 1).

Within each study plot, all vascular plant and bryophyte species were recorded, and their abundance was estimated using the Braun–Blanquet cover and abundance scale [55]. The topography was characterised based on microtopography properties (hummock cover, water table position, and surface water presence). Each study plot was assigned to the types of topography following [48], with slight modifications:

1. Lawns with negligible microtopography and a high level of surface water that reaches at least half of the bryophyte cover. This usually occurs in flooded areas adjacent to lakes, rivers, or streams.
2. Wet lawns (surface water slightly above the peat layer) with shallow hollows.
3. Dry lawns (surface water below peat level) with negligible microtopography.
4. Hummocky areas (hummocks cover up to 50% of the area).
5. Highly hummocky areas (more than 50%) interfacing with deep hollows or water flows.

Additionally, the percentage of hummock cover was evaluated.

Water samples were collected from each plot for hydrochemical analysis. In situ measurements of pH and electrical conductivity were taken using a portable multiparameter device (Multi 350i, WTW, Troistedt, Germany). For chemical analysis, water samples were collected in autumn and frozen until laboratory processing. Concentrations of calcium (Ca^2+^), magnesium (Mg^2+^), iron (Fe^3+^), nitrate (NO_3_^−^), ammonium (NH_4_^+^), potassium (K^+^), and phosphate (PO_4_^3−^) were determined.

Chemical analyses were conducted at the accredited laboratory CJSC “EKOMETRIJA” using standardised ISO and EN ISO methods: Fe^3+^ (ISO 6332:1995), K^+^ (ISO 9964-3:1998), NH_4_^+^ (ISO 7150-1:1998), NO_3_^−^ (ISO 7890-3:1998), PO_4_^3−^ (EN ISO 6878:2004), Ca^2+^ (ISO 6058:2008), and Mg^2+^ (ISO 6059:2008).

### 2.3. Data Analysis

Vascular plants and bryophyte groups were distinguished using the TWINSPAN (Two-Way Indicator Species Analysis) algorithm [56], as implemented in the JUICE software. The dataset consisted of species percentage cover values, with pseudo-species cut levels set at 0, 5, 10, and 20%. The classification was terminated at the third cluster level to ensure the resulting groups represented ecologically interpretable floristic units. Species with a fidelity (*φ* coefficient) exceeding 30% were considered indicator species within each group. The two main TWINSPAN clusters were labelled I–II, with B for bryophytes and H for vascular plants. Groups were numbered 1–11 and similarly marked (e.g., 3B, 5H) for clarity and cross-referencing.

Differences in ecological parameters (hummock cover, pH, topography, conductivity, and concentrations of Ca^2+^, Mg^2+^, Fe^3+^, NO_3_^−^, NH_4_^+^, K^+^, and PO_4_^3^) between plant (IH–IIH) and bryophyte (IB-IIB) clusters identified by the TWINSPAN method were assessed using the Mann–Whitney U test, and differences in the distribution of topography forms were assessed using the Chi-square test. Meanwhile, variations in ecological parameters between vascular plant (1H–11H) and bryophyte (1B–10B) groups were tested using the Kruskal–Wallis test followed by Dunn’s post hoc test. PERMANOVA was used to determine whether the bryophyte and vascular plant groups differed significantly in terms of measured environmental variables. The analysis included species groups identified through classification and a set of measured ecological parameters. Principal component analysis (PCA) was used to identify the main environmental components driving the distribution of vascular plant and bryophyte groups. Components were retained based on the Kaiser criterion, which recommends keeping components with eigenvalues greater than or equal to one [57]. PERMANOVA and PCA were conducted using the PAST statistical software, version 5 [58]. To meet the assumptions of PCA, ecological variables that deviated from a normal distribution (assessed using the Kolmogorov–Smirnov test) were log_10_-transformed to reduce skewness and approximate normality. The Chi-square test was used to determine the distribution differences between groups of bryophytes and vascular plants. Any *p*-value less than 0.05 was regarded as statistically significant.

The taxonomic nomenclature followed the checklist of bryophytes of Europe [59] and the World Checklist of Vascular Plants [60].

## 3. Results

The results of the TWINSPAN in the herb layer illustrated eleven interpreted groups of species (Appendix A). The first cluster (IH), covering groups 1H–5H of the dataset, separated plots with *Carex lepidocarpa* from the second cluster (IIH), which was mostly characterised by *Carex rostrata* and *Agrostis stolonifera* (Appendix A).

Group 1H was characterised by high species richness, in addition to species characteristic to cluster IH (*C. lepidocarpa*, *C. panicea*, etc.), including those typical of cluster IIH, such as *Agrostis stolonifera*, *Carex rostrata*, and *Festuca rubra*, which indicates a mixed composition. Similarly, group 6H, assigned to cluster IIH, also exhibited high species diversity and included species from both clusters. However, unlike group 1H, group 6H was much more frequent and abundant in sedges (*Carex dioica*, *C*. *lepidocarpa*, *C*. *nigra*, *C*. *panicea*, *C*. *rostrata*) and shrubs such as *Betula pubescens* and *Salix* sp. Group 2H was dominated by low graminoids, especially *Eleocharis quinqueflora*, while in group 3H, high constancy was demonstrated by *Carex panicea*. The stands of groups 4H and 5H represented vascular plant compositions with *C. lasiocarpa*. However, in group 4H, *C. lasiocarpa* was more strongly associated with other sedges such as *C. panicea* and *C. limosa*, whereas group 5H showed a greater presence of *Trichophorum alpinum* and shrubs (*Betula pubescens*, *Salix* sp.).

The second TWINSPAN cluster IIH separated six vegetation groups (6H–11H groups). Group 7H was characterised by the dominance of *Eriophorum angustifolium*, occurring alongside associated species such as *Agrostis stolonifera*, *Carex diandra*, and *Caltha palustris*. Groups 8H and 9H were both characterised by pronounced hummock formation, suggesting drier fen microtopography. Group 8H included typical mesotrophic fen species such as *Carex limosa*, *Drosera rotundifolia*, and *Lysimachia thyrsiflora*, indicating more open and structurally diverse habitats. By contrast, tall, nutrient-demanding wetland species such as *Phragmites australis*, *Typha latifolia*, and *Carex diandra* occurred exclusively in group 9H. Group 10H included a relatively diverse set of indicators and frequent species such as *Agrostis stolonifera*, *Calla palustris*, *Caltha palustris*, *Cardamine pratensis*, *Eriophorum angustifolium*, *Festuca rubra*, *Parnassia palustris*, *Salix rosmarinifolia*, and *Silene flos-cuculi*. Group 11H, while overlapping in some floristic elements (e.g., *Agrostis stolonifera*, *Caltha palustris*) with group 10H, is distinguished by the presence and higher abundance of *Carex diandra*, *C. limosa*, *Poa trivialis*, and *Stellaria palustris*.

Bryophyte vegetation was classified into two main clusters (IB-IIB) at the first level (TWINSPAN). The IB cluster comprised bryophyte communities with *Scorpidium cossonii* and *Campylium stellatum*. The IIB cluster was internally more heterogeneous and primarily associated with species *Calliergonella cuspidata* and *Plagiomnium ellipticum* (Appendix B).

The first two bryophyte groups (1B and 2B) represented compositions with *Scorpidium cossonii*; only group 1B differed from 2B in that it had segments of *Sphagnum contortum* and *Cinclidium stygium*, *Calliergon giganteum*, and *Polytrichum strictum*, while the bryophyte layer of group 2B was dominated by *Scorpidium cossonii*. In group 3B, together with *Scorpidium cossonii* and *Campylium stellatum*, the bryophyte layer was formed by *Plagiomnium elatum* and fragments of *Tomentypnum nitens*, *Shagnum teres*, *Aulacomnium palustre*, and *Scorpidium scorpioides.* The stands of groups 4B and 5B represented different species compositions, with the dominant species *Cinclidium stygium* and *Scorpidium scorpioides* in the bryophyte layer. The main difference between these groups lay in their species composition: Group 4B was characterised by the presence of *Drepanocladus trifarius*, *Aulacomnium palustre*, *Aneura pinguis*, and *Mesia triquetra* and a higher abundance of *Campylium stellatum* compared to *Cinclidium stygium*. By contrast, group 5B was notably rich in *Cinclidium stygium* and *Scorpidium scorpioides*. Group 6B exhibited high species richness, comprising species characteristic of the first cluster, including *Scorpidium cossonii* and *Campylium stellatum*, alongside *Aulacomnium palustre* and *Sphagnum teres*, with occasional occurrences of *Hamatocaulis vernicosus*, *Calliergon giganteum*, and *Climacium dendroides*. By contrast, groups 7B and 8B both hosted *Hamatocaulis vernicosus*, *Marchantia polymorpha*, and *Plagiomnium ellipticum*. However, group 8B showed greater species richness, including *Brachythecium mildeanum* and *Tomentypnum nitens*, while *Calliergon giganteum* was found only in group 7B. Groups 9B and 10B were distinguished by *Sphagnum* sp., *Paludella squarrosa*, and *Tomentypnum nitens*, while group 10B was notable for the abundance of *Helodium blandowii*.

The Mann–Whitney U test revealed significant differences in environmental variables between the two vascular plant clusters (Table 2). Specifically, pH and the concentrations of Mg^2+^, Fe^3+^, NH_4_^+^, PO_4_^3−^, and K^+^ differed significantly, with most of these elements being more abundant in cluster IIH. Similarly, when comparing the corresponding bryophyte clusters, significant differences were observed in hummock cover, pH (with cluster IB being more alkaline), and the concentrations of Fe^3+^, K^+^, Mg^2+^, and PO_4_^3−^. As in the vascular plant dataset, these variables were generally more abundant and exhibited broader ranges within cluster IIB of the bryophyte groups.

**Figure 2:**
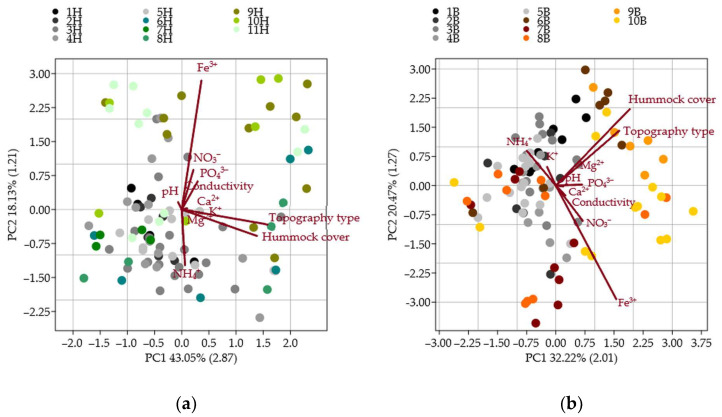
Principal component analysis (PCA) of environmental variables (hummock cover, topography type, pH, conductivity, and concentrations of Ca^2+^, Fe^3+^, K^+^, Mg^2+^, NH_4_^+^, NO_3_^−^, and PO_4_^3−^) influencing the distribution of vascular plant (1H–11H) (**a**) and bryophyte (1B–10B) (**b**) groups; each colour represents a different group based on components (PC) with eigenvalues greater than one (eigenvalues shown in parentheses on axes).

The PCAs were conducted exclusively between vascular plant groups and bryophyte groups to identify the key environmental variables influencing species composition (Table 3). Among the ten measured environmental factors, hummock cover, topography, and iron concentration consistently emerged as the most influential in explaining the variation within both vascular and bryophyte groups (Figure 2). Although the same environmental factors were significant across all groups, their relative contributions differed, i.e., hummock cover had the most decisive influence on bryophyte communities, while topography more strongly affected vascular plant species distribution.

**Figure 3:**
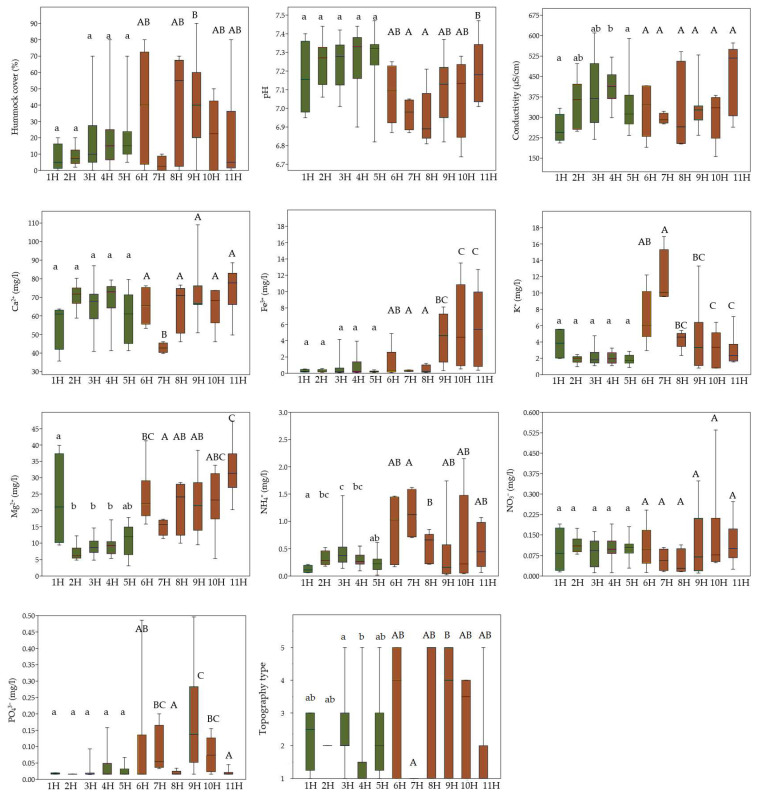
Comparison of ecological variables (hummock cover; pH; conductivity; concentrations of Ca^2+^, Fe^3+^, K^+^, Mg^2+^, NH_4_^+^, NO_3_^−^, and PO_4_^3−^; and topography types) among vascular plant groups (1H–11H) using boxplots, showing significant differences in environmental conditions. Colours represent different clusters assigned to each group. Lowercase letters indicate statistically significant differences among groups within cluster IH, while uppercase letters indicate differences among groups within cluster IIH. Statistical differences were tested using the Kruskal–Wallis test followed by Dunn’s post hoc test (*p* < 0.05).

**Figure 4:**
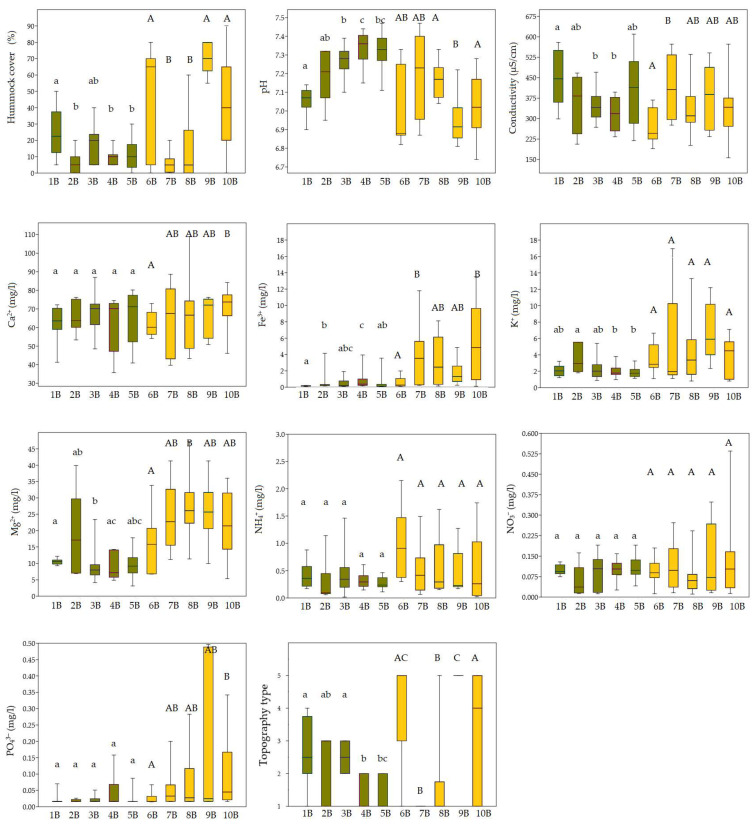
Comparison of ecological variables (hummock cover; pH; conductivity; concentrations of Ca^2+^, Fe^3+^, K^+^, Mg^2+^, NH_4_^+^, NO_3_^−^, and PO_4_^3−^; and topography type) among bryophyte groups (1B–10B) using boxplots, showing significant differences in environmental conditions. Colours represent different clusters assigned to each group. Lowercase letters indicate statistically significant differences among groups within cluster IB, while uppercase letters indicate differences among groups within cluster IIB. Statistical differences were tested using the Kruskal–Wallis test followed by Dunn’s post hoc test (*p* < 0.05).

Environmental parameters associated with the five bryophyte groups (1B–5B) and five vascular plant groups (1H–5H) generally reflect stable, moderately calcium-rich conditions with limited variation in key ecological factors. However, among vascular plant groups, group 1H stood out due to lower conductivity and elevated concentrations of magnesium and potassium. By contrast, bryophyte groups (1B–5B) showed more apparent ecological differentiation, particularly in terms of pH and hummock cover, with group 2B exhibiting notably higher levels of magnesium and potassium (Figure 3 and Figure 4).

In the case of the vascular plant groups from the second cluster IIH, the species-rich group 6H, characterised by greater hummock cover, preferred a slightly higher availability of NO_3_^−^, NH_4_^+^, Mg^2+^, and K^+^. Patches with *Eleocharis quinqueflora* (group 7H) thrived in mineral-rich, wet, and slightly more acidic conditions, exhibiting lower Ca^2+^, Fe^3+^, and NO_3_^−^ but the highest concentrations of K^+^ compared to other IIH groups. Groups 8H and 9H were associated with the development of hummocks and mineral-rich conditions. On the other hand, group 9H was more relevant to higher Fe^3+^ and PO_4_^3−^ concentrations. Group 10H shared similar environmental conditions with group 11H, both being associated with rich and moist habitat features. However, group 11H was distinguished by slightly wetter conditions, higher electrical conductivity, and lower pH values as well as lower PO_4_^3−^ (Figure 3).

Vegetation patterns across the identified *Calliergonella cuspidata* and *Plagiomnium ellipticum* groups (6B–10B) were strongly influenced by microtopography and water chemistry. Group 6B was characterised by drier microsites and low Fe^3+^ and conductivity, but high NO_3_^−^ and NH_4_^+^. By contrast, groups 7B and 8B represented wetter, base-rich areas with higher Fe^3+^, Mg^2+^, and conductivity and were ecologically similar. Groups 9B and 10B were marked by hummock development and high concentrations of Mg^2+^, Fe^3+^, and PO_4_^3−^, with group 9B exhibiting especially elevated K^+^ and PO_4_^3−^ (Figure 4).

PERMANOVA results revealed significant differences among bryophyte groups based on the measured environmental variables, indicating that bryophyte communities exhibited greater ecological differentiation compared to vascular plant groups (Table 4 and Table 5).

**Figure 5:**
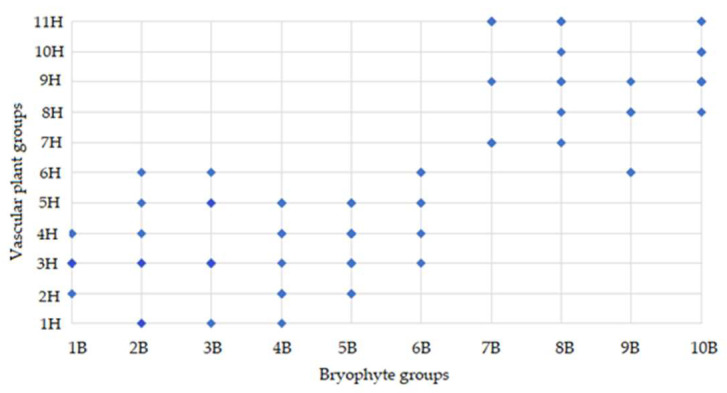
Binary association matrix illustrating ecological group-level relationship between vascular plant (1H–11H) (Appendix A) and bryophyte groups (1B–10B) (Appendix B) (based on co-occurrence in study sites).

The joint distribution of bryophyte and vascular plant groups highlights apparent asymmetries in the ecological amplitude and habitat specificity of vascular plant groups of different clusters. Bryophyte groups 1B–5B were closely related to vascular plant groups of the IH cluster, while 7B–10B bryophyte groups were with the vascular groups from the IIH cluster (Figure 5).

## 4. Discussion

### 4.1. Main Characteristics of Bryophyte and Vascular Plant Covers in the Studied Fens

As in the northeastern part of Poland [49] neighbouring Lithuania, the communities we studied, based on vascular plant cover, are divided into two clusters based on the distribution of *Carex lepidocarpa* and *C. rostrata*. Concerning the *C. lepidocarpa* cluster, most of the diagnostic species (*C. panicea*, *Drosera anglica*, *Eleocharis quinqueflora*, *Eriophorum latifolium*, *Peucedanum palustre*) are the same [49]. Additionally, the communities we described are also characterised by the presence of *Trichophorum alpinum*, which is found in various parts of Europe, growing together with previously mentioned species [61]. The species *Epipactis palustris* and *Cirsium palustre*, indicated as diagnostic for the *Carex lepidocarpa* cluster [49], were found equally distributed in both clusters. In the newest synthesis of fen alliances of Europe, only *C. panicea* and *Eriophorum latifolium* are indicated as diagnostic for *Caricion davallianae* communities. The other species mentioned have no diagnostic role in any of the alliances.

In addition to the species *Agrostis stolonifera*, *Carex diandra*, *Epilobium palustre*, *Ranunculus lingua*, *Rumex acetosa*, and *Silene flos-cuculi*, which are provided as characteristic of Poland fen communities indicated by *Carex rostrata* [49], we found that this cluster is also marked by *Caltha palustris*, *Festuca rubra*, *Galium uliginosum*, and *Myosotis scorpioides*. Usually, *Carex rostrata* stands belong to *Caricetum rostratae* (*Caricion rostratae*, Magnocaricetalia) [62]. The water level is an essential factor for the growth of *C. rostrata* shoots, especially during early summer when most of the length increment occurs [63]. *Carex rostrata* and plants with high stem porosity are adaptive to growing in areas with high Fe^3+^ concentrations [64]. *Carex rostrata* usually has high fidelity in the habitats of alliances *Stygio-Caricion limosae*, *Sphagno-warnstorfii Tomentypnion*, *Saxifrago-Tomentypnion*, and *Sphagno caricion canescentis* [40].

Unlike previous research [49], which was focused exclusively on bryophyte carpets, our study incorporated plots displaying a variety of microtopographic features. This allowed us to reveal a greater diversity of bryophytes in both clusters, including *Cinclidium stygium*, *Drepanocladus trifarius*, *Meesia triquetra*, *Paludella squarrosa*, *Helodium blandowii*, and *Sphagnum* species. Despite the greater diversity of bryophytes, the two main clusters featured similar species to those in the Polish study [49].

Similar to the vascular plant communities from the IH cluster, bryophytes of the IB cluster, indicated by *Campylium stellatum* and *Scorpidium cossonii*, also prefer rich fens characterised by stable or only slightly fluctuating water tables and higher pH values, which is consistent with the Polish findings [49].

In the compendium of fen alliances [40], it is indicated that *C. stellatum* reaches the highest frequency in *Caricion davallianae*, *Caricion atrofusco saxatilis*, and *Sphagno warnstorfii-Tomentypnion* alliances; however, the diagnostic role of *Scorpidium cossonii* in this compendium is not defined, as the species is integrated into an ecologically wider, especially according to habitat acidity [65], assemblage aggr. *Drepanocladus revolvens.*

*Hamatocaulis vernicosus*, *Marchantia polymorpha*, and *Plagiomnium ellipticum*, the diagnostic species of the IIB cluster, also distinguish one of the vegetation groups in the Polish study [49]. It is important to emphasise that our research revealed *Calliergonella cuspidata* and *Plagiomnium ellipticum* to play a more significant role in distinguishing the two clusters than the other species mentioned. *C. cuspidata* and *P. ellipticum* usually thrive within the same general habitat but occupy different micro-niches. *Calliergonella cuspidata* is typically found in more open habitats with higher nutrient content, such as base-rich fens. It prefers moderately moist-to-wet substrates with stable hydrological conditions but can tolerate a range of moisture regimes [61,66,67]. In some studies [16,19,68], *C. cuspidata* is commonly recognised as an indicator species of both eutrophication and drying conditions in fen ecosystems. *Plagiomnium ellipticum* prefers shaded areas with stable hydrological conditions. It is often found in slightly wetter areas, forming dense patches in depressions or shallow hollows where water accumulates [65,69]. These species co-occur not because they are ecologically similar, but because they have overlapping tolerance ranges that enable them to thrive under specific environmental conditions.

Our results are consistent with the findings by Pawlikowski et al. [49], showing a strong co-occurrence between *Plagiomnium ellipticum* and *Hamatocaulis vernicosus* and suggesting that these species may share similar ecological preferences. Meanwhile, *Calliergonella cuspidata* has been found by Pawlikowski [49] to occur with similar frequency across two floristically distinct rich fen types. By contrast, our study, as well as previous studies [48,61,70,71], suggest that *C. cuspidata* is less prevalent in fens dominated by *Scorpidium cossonii* in the bryophyte layer. This may be attributed to the differing ecological requirements of the two species. *Calliergonella cuspidata* prefers more nutrient-rich habitats and is better adapted to dynamic fen environments than *Scorpidium cossonii* [19,68].

Although we analysed bryophyte and plant diversity separately, our study confirms the two groups of fen vegetation found in Poland [49] and provides further evidence of their ecological differences at a broader geographic scale. Additionally, in line with the pH differences between the plant clusters ascertained by Pawlikowski et al. [49], we observed significant differences in pH and the amounts of Ca^2+^, Fe^3+^, Mg^2+^, K^+^, NO_3_^−^, and PO_4_^3−^ in their habitats. This may be associated with the regional characteristics of the fens, which, in most cases, are fed by mineral-rich springs (especially iron-rich ones) [72]. Usually, *Carex lepidocarpa* is provided as an indicator of calcareous fens. However, calcium levels were higher in *Carex rostrata* than in *C. lepidocarpa* communities in the fens we studied. This confirms the need to obtain reliable data from a wider area of Eastern Europe to specify the composition, ecology, and distribution pattern of the plant communities formed by *C. rostrata* and *C. diandra* [49].

### 4.2. Mosaic Structure of Vascular Plant Cover

The two main clusters of vascular plants and bryophytes reflect the regional peculiarities of Lithuanian fens. The further distinguished groups of species pools reflect the mosaic structure of fen communities, which may be defined by local ecological conditions.

The distributions of vascular plant groups are in accordance with the insight that water level is one of the important axial factors influencing the distribution of vascular plants in fen communities, and it is the major determinant of small-scale patterns of species distribution [37]. Water level, in our study, is indirectly presented as topography, and is a key factor in determining the mosaic nature of the plant communities in the *C. lepidocarpa* cluster, where most of the chemical parameters are similar. The indicator species for the vegetation pools in this cluster are usually *Eleocharis quinqueflora*, *Carex panicea*, and *C. lasiocarpa.* The patches dominated by *Eleocharis quinqueflora* occur at water-saturated sites of calcareous fens [73]. *Carex panicea* is a characteristic species of fen meadows. The investigations of drained fens in Sweden show the *C. panicea* peak at five to ten years after drainage [23]. Thus, the extension of such communities in the fens may be an indication of their encroachment. According to [74], plant communities with *C. lasiocarpa* and calciphilous species represent the wetter face of *Caricion davallianae*. The wetter characteristic in our study is also indicated by the species growing together: *Carex diandra*, *C. limosa*, *Comarum palustre*, etc.

Vegetation heterogeneity may be enhanced by an expanding shrub and tree cover, which is often linked to a drop in water levels and hummocky character of the area [23]. The occurrence of shrubs (*Salix rosmarinifolia*), meadow species (*Rumex acetosa*, *Silene flos-cuculi*, and *Festuca rubra*), and wetland plants (e.g., *Carex limosa*, *Calle palustris*, *Caltha palustris*) in the same vegetation pool reflects the co-occurrence of hummocks and the hollows.

When analysing the differences between the two clusters as well as within IIH, we found that iron content is also a key factor in determining the mosaic structure of their vegetation. The content of this toxic metallic element can influence patterns of small-scale vegetation [37,38]. Extremely high iron was fixed within the vegetation patches of the 9H, 10H, and 11H groups. However, many species characteristic of these groups, such as *Agrostis stolonifera*, *Caltha palustris*, and *Calle palustris*, are also common in habitats with lower iron content (group 7H). For plants inhabiting fens fed by iron-rich waters, high iron concentrations are probably not necessary but are tolerable. We found that, in addition to these species, the patches with low iron and calcium were dominated by *Eriophorum angustifolium.* Usually, *E. angustifolium* communities are basic elements of mire vegetation in *Sphagnum* peatlands [75]. However, the species can tolerate a wide range of pH values (3.7–6.8), nitrates, base deficiency, and iron [76]. The high amount of potassium recorded by us in the patches dominated by *E. angustifolium* seems to be limited by potassium as in the case of *E. vaginatum* [77]. Another distinguishing feature of this group habitat is the high levels of ammonium coupled with low amounts of nitrates. This may be related to the high water levels in the habitat, which could be affecting the reduction processes [18]. Two groups indicated by *Carex limosa* (8H and 11H) are distinguished by a greater extent of surface water or the presence of hollows, as well as by a low amount of phosphorus content. The species exhibits characteristics (a low fresh shoot ratio) that may help it grow under low phosphate conditions [78].

It is important to note that two groups (1H and 6H) are classified in different clusters, although they share diagnostic species that are specific to both. Group 6H, attributed to the *C. rostrata* cluster, is defined by the presence of *C. dioica* and *C. nigra*. The latter is a diagnostic species of the *Caricion fuscae* alliance and occurs in habitats with moderate-to-low calcium content [40]. Although our studies do not indicate any differences in calcium content between these groups, group 6H is closer to cluster IIH in terms of other parameters.

### 4.3. Bryophyte Pools and Their Ecological Preferences

As with vascular plant groups, the heterogeneity of rich fens, as manifested through features such as water levels, hummocks, and hollows, shapes the distribution patterns of bryophyte groups observed in our study. Patches indicated by *Scorpidium scorpioides* and *Cinclidium stygium*, along with inclusions of *Drepanocladus trifarius*, *Meesia triquetra*, and *Aneura pinguis*, as well as bryophyte groups dominated by species such as *Hamatocaulis vernicosus* and *Calliergon giganteum*, were more strongly tied to stable, waterlogged hollows or lawns, where water availability remains consistently high. This finding is consistent with previous experiments, which have shown that these species exhibit high vitality under prolonged waterlogged conditions [18]. By contrast, patches dominated by *Scorpidium cossonii* and *Campylium stellatum* appear to indicate that the water table lay somewhat deeper.

Groups 6B, 9B, and 10B represent bryophyte species that actively form hummocks. Likewise, *Paludella squarrosa* and *Helodium blandowii*, typically considered indicators of wetter environments [65], were frequently associated with low hummocks in relatively dry microsites in our study, suggesting a broader ecological amplitude and potential adaptation to spatial moisture variability [67]. Hummock formation by bryophytes, especially those with high water-holding capacity and dense canopy structure, not only reduces desiccation stress but also contributes to fen stability or, conversely, promotes ecological shifts by altering hydrological feedback [79,80,81]. Moreover, our findings support previous studies e.g., [82,83,84], indicating that increased hummock abundance is often linked to declining pH values. Wet carpets dominated by species like *Hamatocaulis vernicosus* and *Scorpidium scorpioides* occur under slightly alkaline conditions, while areas with more hummock-forming species are associated with more acidic habitats.

It is also crucial to highlight the role of iron content in shaping the spatial patterns of bryophytes. In fen habitats, iron plays a crucial role in phosphorus cycling, which in turn affects plant growth and overall ecosystem health [64]. Patches dominated by *Hamatocaulis vernicosus* were commonly found in areas with high iron concentrations, although this species showed tolerance to a wide range of iron levels [48]. Although the presence of *H. vernicosus* is associated with higher phosphorus levels [49], our previous research [48] and the current study do not show that these species habitats are distinguished by their phosphorus content. Additionally, high iron levels were also recorded in hummocky group 10B. At the same time, phosphorus exhibited a notably wide range of values in group 9H—with frequent patches of *Palludella squarrosa* in the bryophyte layer. This variability in phosphorus and iron concentrations warrants further detailed analysis, as it may reflect localised environmental heterogeneity. Potential factors include differences in redox conditions, organic matter decomposition, and microtopography (e.g., presence of hollows and hummocks) [18,85].

Our study found high levels of base cations (Ca^2+^, Mg^2+^, and K^+^) in rich fens, although these factors appeared to have only a moderate influence on the distribution of bryophyte groups. Unlike some reports linking *Scorpidium cossonii* to higher calcium levels [72,86,87], we observed no significant calcium differences, but the ratio of calcium to magnesium and potassium appeared more relevant [84]. Additionally, hummocky areas with patches of *Paludella squarrosa* and *Helodium blandowii* exhibited notably higher potassium levels, ranging from five to six mg/L. In terms of NH_4_^+^ concentrations, they were notably higher and more variable, with several sites exceeding two mg/L, indicating potential organic pollution [88] or reduced oxygen conditions in groups 6B and 10B. These findings suggest that the bryophyte communities observed in these groups may be subjected to elevated environmental stress or anthropogenic disturbance [50].

### 4.4. Vascular and Bryophyte Groups as Indicators of Habitat Conditions

Our study indicates that the distribution of bryophyte and vascular plant groups is primarily influenced by the same factors: topography, number of hummocks, and iron content. However, vascular plant groups exhibit a wider range of overlaps with ecological conditions compared to bryophyte groups. Vegetation patches defined by the same vascular plant assemblages or dominant plant species may cover bryophyte pools with a different species composition. Vascular plants are less sensitive to microtopographic or hydrological changes than are bryophytes due to their more efficient water conducting system. They can penetrate deeper into peat layers and are thus expected to sustain water uptake and, consequently, transpiration [89,90]. In addition, the development of vascular plants, such as sedges, may be determined by competition between species due to their different life traits. The gaps in the vegetation that are necessary for seed germination and seedling establishment are often unavailable in dense stands of rhizomatous, clonal species [91]. Therefore, initial changes in environmental conditions in fens may be more accurately identified by changes in the composition of bryophytes than by vascular plants. As Hajek et al. [19] stated, shifts in bryophyte species composition also indicate undesirable successional changes in fens. Conversely, the diversity of bryophytes may be linked to slight differences in micro-relief that persist despite general changes in the fen’s hydrology. It is essential to note that, when evaluating changes in habitat conditions, identifying the exact species, not just complexes of species, is crucial.

Nonetheless, there is significant overlap between specific plant and bryophyte groups within the clusters, while the differences between the clusters are pronounced. The exclusion of intermediate groups between the respective clusters of vascular plants (6H) and bryophytes (6B) makes this particularly striking. On one side, there are the fen communities characterised by *Carex lepidocarpa* and *Scorpidium cossonii* and, on the other, those characterised by *Carex rostrata* and *Hamatocaulis vernicous*. This shows once again that such fens are common in the region, which is transitional between the boreal and continental regions. There are two distinct types of fens, which are significantly different in their species composition from those described in Central Europe [48,49]. We agree with Pawlikowski et al. [49] that the fens we described do not exactly fit the scheme according to the poor-rich gradient provided for Central Europe [68]. Most importantly, it has no place for the plant communities characterised by the most typical species, including *Carex rostrata*, *C. diandra*, *Agrostis stolonifera*, *Caltha palustris*, and *Hamatocaulis vernicosus* in the moss cover. While such fen habitats are abundant in calcium and minerals, they do not contain calcareous species, as has also been reported by Pawlikowski [49]. Although *H. vernicosus* is listed as a characteristic of extremely rich fen species in the scheme [68], these fens are also characterised by the presence of *Scorpidium cossonii* and *Campylium stellatum*. The only bryophyte group that harbours both *Hamatocaulis vernicosus* and *Scordidium cossonnii* and meets the group’s criteria for calcareous vascular plants is group 6B. Therefore, there is still scope for debate on how the fens in our region should be divided.

## 5. Conclusions

Our research identified two ecologically and floristically distinct plant communities that are representative of regional fen ecosystems. Such data are highly relevant for the fen classification efforts at both the national and European scales. The mosaic distribution of vegetation, observed in both bryophyte and vascular plant layers, is primarily shaped by local hydrological regimes, microtopographical variation, and the amount of iron. Given their greater ecological differentiation, bryophyte assemblages serve as particularly sensitive indicators and should be given special attention in conservation planning and habitat management.

## Figures and Tables

**Table 1:** List of investigated fens, their locations, and number of study plots.

Study Site no.	Fen Name	Administrative Unit	Coordinates (WGS-84)		Number of Study Plots
			Lat.	Lon.	
1	Acintas	Švenčioniai District	55.00198	25.97825	4
2	Bražuolė	Trakai District	54.70632	24.88171	11
3	Burgis	Plateliai District	56.02479	21.92462	15
4	Čiaunas	Zarasai District	55.77684	25.87725	7
5	Pelesa	Varėna District	56.01642	21.79576	4
6	Velėnija	Plateliai District	56.01105	21.82575	3
7	Juodupis	Plateliai District	56.06659	21.84840	7
8	Pravalas	Molėtai District	54.95740	25.67367	8
9	Siberija	Plateliai District	56.03012	21.81525	9
10	Skerdzimų pieva	Varėna District	54.01560	24.29427	10
11	Smalvos	Zarasai District	55.61854	26.35358	8
12	Šeirė	Plateliai District	56.05417	21.83646	2
13	Šillėnai	Vilnius District	54.73043	25.03369	6
14	Tyras	Plungė District	56.004667	21.794748	3
15	Fen by the Verknė River	Trakai District	54.54754	24.50527	4

**Table 2:** Statistical comparisons of environmental parameters (hummock cover; pH; conductivity; concentrations of Ca^2+^, Fe^3+^, K^+^, Mg^2+^, NH_4_^+^, NO_3_^−^, and PO_4_^3−^; and topography type) between vascular plant (I-IIH) and bryophyte (I-IIB) clusters. All environmental parameters were analysed using the Mann–Whitney U test, while differences in the distribution of topography forms were assessed using the Chi-square test. Significant differences (*p* < 0.05) are highlighted in bold.

Parameters	Vascular Plants			Bryophytes		
	I H (n = 56) (Mean ± SD)	II H (n = 42) (Mean ± SD)	*p*	I B (n = 54) (Mean ± SD)	II B (n = 44) (Mean ± SD)	*p*
Hummock cover (%)	17.89 ± 18.46	29.92 ± 29.36	0.30	13.74 ± 11.05	34.47 ± 30.75	**0.02**
pH	7.24 ± 0.02	7.08 ± 0.17	**0.00**	7.26 ± 0.01	7.08 ± 0.03	**0.00**
Conductivity (µS/cm)	371.71 ± 14.02	354.67 ± 113.84	0.35	376.22 ± 104.8	349.91 ± 112.60	0.15
Ca^2+^ (mg/L)	64.83 ± 10.92	66.63 ± 14.19	0.64	64.65 ± 11.10	66.77 ± 13.86	0.59
Fe^3+^ (mg/L)	0.54 ± 0.92	3.51 ± 3.8	**0.00**	0.53 ± 0.91	3.40 ± 3.79	**0.00**
K^+^ (mg/L)	2.16 ± 1.01	4.85 ± 3.72	**0.00**	2.22 ± 1.11	4.64 ± 3.70	**0.00**
Mg^2+^ (mg/L)	10.21 ± 5.89	24.17 ± 9.40	**0.00**	10.64 ± 6.27	23.01 ± 13.86	**0.00**
NH_4_^+^ (mg/L)	0.34 ± 0.26	0.63 ± 0.56	0.13	0.35 ± 0.28	0.60 ± 0.55	0.14
NO_3_^−^ (mg/L)	0.09 ± 0.03	0.10 ± 0.10	0.30	0.09 ± 0.03	0.10 ± 0.10	0.33
PO_4_^3−^ (mg/L)	0.02 ± 0.02	0.08 ± 0.12	**0.00**	0.02 ± 0.02	0.08 ± 0.11	**0.00**
Topography type						
1	15 (26.8%)	22 (52.4%)	**0.00**	16 (29.6%)	21 (47.7%)	**0.00**
2	23 (41.1%)	-		23 (42.6%)	-	
3	12 (21.4%)	2 (4.8%)		13 (24.1%)	1 (2.3%)	
4	2 (3.6%)	6 (14.3%)		2 (3.7%)	6 (13.6%)	
5	4 (7.1%)	12 (28.6%)		-	16 (36.4)	

**Table 3:** PCA loadings of environmental parameters (hummock cover, topography type, pH, conductivity, and concentrations of Ca^2+^, Fe^3+^, K^+^, Mg^2+^, NH_4_^+^, NO_3_^−^, and PO_4_^3−^) and species groups (vascular plants and bryophytes) across study sites (n = 98). Loadings in bold indicate high contributions (|loading| > 0.4).

Parameters	Vascular Plant Groups		Bryophyte Groups	
	PC 1	PC 2	PC 1	PC 2
Hummock cover (%)	**0.63**	−0.17	**0.60**	**0.49**
Topography type	**0.72**	−0.09	**0.51**	0.34
pH	−0.03	0.05	0.03	−0.03
Conductivity (µS/cm)	0.03	0.08	0.08	−0.06
Ca^2+^ (mg/L)	0.02	0.04	0.06	−0.05
Fe^3+^ (mg/L)	0.17	**0.84**	**0.48**	**−0.72**
K^+^ (mg/L)	0.09	−0.01	−0.07	0.11
Mg^2+^ (mg/L)	0.04	0.01	0.06	−0.01
NH_4_^+^ (mg/L)	0.03	−0.37	−0.23	0.22
NO_3_^-^ (mg/L)	0.09	0.26	0.21	−0.22
PO_4_^3-^ (mg/L)	0.13	0.18	0.21	0.01
Eigenvalue	2.87	1.21	2.01	1.27
Explained % of variance	43.05	18.13	32.22	20.47

**Table 4:** Pairwise vascular plant group differences based on PERMANOVA of environmental parameters (hummock cover, topography type, pH, conductivity, and concentrations of Ca^2+^, Fe^3+^, K^+^, Mg^2+^, NH_4_^+^, NO_3_^−^, and PO_4_^3−^). Significant differences (*p* < 0.05) are highlighted in bold.

Vascular Plant Groups	1H	2H	3H	4H	5H	6H	7H	8H	9H	10H	11H
**1H**		**0.04**	**0.01**	**0.01**	0.06	0.23	**0.02**	0.29	**0.00**	0.11	**0.00**
**2H**	**0.04**		0.35	0.12	0.13	**0.00**	**0.00**	**0.00**	**0.00**	**0.00**	**0.00**
**3H**	**0.01**	0.35		**0.02**	0.09	**0.00**	**0.00**	**0.00**	**0.00**	**0.00**	**0.00**
**4H**	**0.01**	0.12	**0.02**		0.10	**0.01**	**0.00**	**0.00**	**0.00**	**0.00**	**0.00**
**5H**	0.06	0.13	0.09	0.10		**0.00**	**0.00**	**0.00**	**0.00**	**0.00**	**0.00**
**6H**	0.23	**0.00**	**0.00**	**0.00**	**0.00**		0.08	0.85	**0.01**	0.19	0.05
**7H**	**0.03**	**0.00**	**0.00**	**0.00**	**0.00**	0.07		0.08	**0.00**	**0.04**	**0.00**
**8H**	0.28	**0.00**	**0.00**	**0.00**	**0.00**	0.85	0.08		**0.00**	0.12	**0.01**
**9H**	**0.00**	**0.00**	**0.00**	**0.00**	**0.00**	**0.01**	**0.00**	**0.00**		0.46	**0.00**
**10H**	0.11	**0.00**	**0.00**	**0.00**	**0.00**	0.19	**0.04**	0.12	0.46		0.32
**11H**	**0.00**	**0.00**	**0.00**	**0.00**	**0.00**	0.05	**0.00**	**0.02**	**0.00**	0.32	

**Table 5:** Pairwise bryophyte group differences based on PERMANOVA of environmental parameters (hummock cover, topography type, pH, conductivity, and concentrations of Ca^2+^, Fe^3+^, K^+^, Mg^2+^, NH_4_^+^, NO_3_^−^, and PO_4_^3−^). Significant differences (*p* < 0.05) are highlighted in bold.

Bryophyte Groups	1B	2B	3B	4B	5B	6B	7B	8B	9B	10B
**1B**		**0.00**	0.21	**0.00**	**0.00**	**0.01**	**0.00**	**0.00**	**0.00**	**0.00**
**2B**	**0.00**		**0.03**	**0.00**	**0.00**	**0.02**	**0.01**	**0.01**	**0.00**	**0.01**
**3B**	0.21	**0.03**		**0.01**	**0.00**	**0.00**	**0.00**	**0.00**	**0.00**	**0.00**
**4B**	**0.00**	**0.00**	**0.01**		0.06	**0.00**	**0.01**	**0.00**	**0.00**	**0.00**
**5B**	**0.00**	**0.00**	**0.00**	0.06		**0.00**	**0.00**	**0.00**	**0.00**	**0.00**
**6B**	**0.02**	**0.02**	**0.00**	**0.00**	**0.00**		**0.00**	**0.00**	0.20	0.12
**7B**	**0.00**	**0.01**	**0.00**	**0.00**	**0.00**	**0.00**		0.97	**0.00**	**0.05**
**8B**	**0.00**	**0.02**	**0.00**	**0.00**	**0.00**	**0.00**	0.97		**0.00**	0.08
**9B**	**0.00**	**0.00**	**0.00**	**0.00**	**0.00**	0.20	**0.00**	**0.00**		0.28
**10B**	**0.00**	**0.01**	**0.00**	**0.00**	**0.00**	0.11	**0.04**	0.08	0.27	

## Data Availability

The data presented in this study are available upon request from the corresponding author. The data are not publicly available due to privacy concerns.

## Appendix A

**Table A1:** Results of TWINSPAN classification showing species presence, frequency, and *φ* coefficients for indicator species across different vascular plant groups (1H–11H). Columns represent vascular plant groups, rows represent species, and each cell contains species frequency with the *φ* coefficient indicated as a superscript. Dominant species were those with a frequency greater than 50% and a *φ* coefficient above 30 (*φ* coefficient expressed as a percentage, i.e., multiplied by 100).

Vascular Plant Groups	1H	2H	3H	4H	5H	6H	7H	8H	9H	10H	11H
No. of relevés	6	6	21	13	12	4	4	5	11	6	10
Species											
*Agrostis canina*	-	-	5	7 ^1.3^	-	-	-	-	-	-	-
*Agrostis stolonifera*	33 ^46.4^	-	-	-	8 ^2.4^	20	100 ^26.2^	40	82 ^7.5^	100 ^26.2^	100 ^26.2^
*Alnus glutinosa*	17 ^14.2^	-	5	7 ^1.3^	17 ^14.2^	17	-	-	9 ^27.7^	-	-
*Andromeda polifolia*	-	17	14	53 ^32.2^	17 ^9.8^	-	-	-	-	-	-
*Angelica sylvestris*	33 ^45.5^	-	10 ^4.1^	-	-	-	-	-	-	-	-
*Betula pubescens*	17	17	29 ^7.1^	15	50 ^30.3^	100 ^66.2^	-	60 ^27.6^	18	0	10
*Calla palustris*	-	-	5 ^20^	-	-	25	50 ^18^	-	36 ^4.8^	67 ^34.1^	10
*Caltha palustris*	50 ^43.3^	-	5	15 ^3.3^	17 ^1.8^	50	100 ^27.3^	60	27	100 ^27.3^	100 ^27.3^
*Cardamine pratensis*	33	50 ^12.8^	19	30 ^2.3^	75 ^36.1^	-	100 ^28.9^	80 ^9.2^	64	100 ^28.9^	80 ^9.2^
*Carex chordorrhiza*	-	-	5	38 ^32.2^	67 ^33.4^	-	-	-	-	-	-
*Carex diandra*	17	33 ^3.4^	19	53 ^27.2^	17	50	100 ^21.7^	80	73	83 ^2.6^	100 ^21.7^
*Carex dioica*	17 ^11.7^	-	5	7 ^5.9^	-	75 ^84.5^	-	-	-	-	-
*Carex flava*	-	-	-	-	-	17	-	-	9	-	-
*Carex lasiocarpa*	33	-	10	85 ^20.2^	100 ^44.8^	-	-	-	-	-	-
*Carex lepidocarpa*	67	83	100 ^18^	92 ^11^	100 ^18^	100 ^82.3^	-	20	-	17	-
*Carex limosa*	17	67 ^9.3^	43	76 ^36.2^	42	-	-	80 ^47.2^	-	17	90 ^56.9^
*Carex nigra*	-	17 ^0.4^	5	23 ^24.8^	-	75 ^72.5^	-	20 ^5.1^	-	-	-
*Carex panicea*	50	17	86 ^30.9^	76 ^20.3^	8	100 ^82.3^	-	20	-	17	-
*Carex rostrata*	83 ^32.9^	-	38	61 ^8^	8	100 ^14.4^	100 ^14.4^	60	100 ^14.4^	83	100 ^14.4^
*Cirsium palustre*	33 ^42.8^	-	5	-	8^1^	75 ^61.2^	-	20	9	17	-
*Cladium mariscus*	17 ^28^	-	-	-	8 ^9.3^	-	-	-	-	-	-
*Comarum palustre*	-	17	33	69 ^32.2^	17	-	-	40 ^59.8^	-	-	-
*Crepis palustris*	-	-	5	-	-	-	-	-	-	-	-
*Dactylorhiza incarnata*	33 ^6.5^	17	43 ^16.1^	46 ^27.2^	8	50 ^31.5^	25 ^4.1^	-	9	33 ^13.2^	10
*Dactylorhiza majalis subsp. baltica*	17 ^37.8^	-	-	-	-	25 ^29.7^	-	20 ^21.2^	-	-	-
*Drosera anglica*	-	83 ^22.7^	76 ^16.3^	46 ^3^	83 ^22.7^	-	-	20 ^41.5^	-	-	-
*Drosera rotundifolia*	-	33 ^39.7^	10 ^1.6^	-	-	50 ^23.8^	-	100 ^74.5^	9	-	-
*Eleocharis quinqueflora*	-	83 ^49.2^	29	30 ^1.3^	17	-	-	20 ^41.5^	-	-	-
*Epilobium palustre*	100 ^66.1^	-	29	7	50 ^17.9^	25	50	100 ^24.9^	100 ^24.9^	83 ^7.3^	100 ^24.9^
*Epipactis palustris*	83 ^23.5^	33	81 ^21.3^	61 ^17^	67 ^8.4^	100 ^16.8^	100 ^16.8^	80	73	83	90 ^3.2^
*Equisetum fluviatile*	83 ^18.6^	17	38	69 ^17.6^	92 ^26.3^	75	100 ^17.3^	100 ^17.3^	100 ^17.3^	67	80
*Equisetum palustre*	-	-	5	-	-	-	-	-	-	-	-
*Eriophorum angustifolium*	50 ^21.7^	17	19	15 ^9.6^	17	75 ^26.8^	100 ^49.3^	20	9	67 ^19.4^	-
*Eriophorum latifolium*	33	-	43 ^8.6^	53	33 ^24.8^	25 ^46.6^	-	-	-	-	-
*Eriophorum gracile*	-	-	-	15 ^7.3^	-	-	-	-	-	-	-
*Eupatorium cannabinum*	50 ^60.8^	-	-	-	8	75 ^58.5^	-	20	-	33 ^13^	-
*Festuca rubra*	50^60^	-	10	-	8	50	25	80 ^14.6^	91 ^24.8^	100 ^33.3^	40
*Galium palustre*	50	33	62 ^6.7^	53 ^22^	42	50	75	60	100 ^24.1^	100 ^24.1^	80 ^2.7^
*Galium uliginosum*	33 ^31.1^	17 ^7.6^	10	-	8	100 ^25.6^	75	80 ^4.8^	64	83 ^8.3^	50
*Juncus articulatus*	-	-	33 ^54.2^	-	-	-	25	-	18	67 ^55.9^	-
*Liparis loeselii*	-	33 ^16.2^	5	7 ^8^	42 ^28.3^	17	-	-	-	17	-
*Lysimachia thyrsiflora*	17 ^14.2^	17 ^14.2^	5	7 ^1.3^	-	25	75 ^34.8^	80 ^39.5^	27	17	-
*Menyanthes trifoliata*	100 ^20.7^	83 ^1.2^	90 ^9.6^	69 ^1.2^	83	75	-	100 ^25.2^	100 ^25.2^	100 ^25.2^	80 ^4.4^
*Myosotis scorpioides subsp. scorpioides*	50 ^57.6^	-	5	-	8	-	100 ^41.1^	60 ^5.2^	82 ^24.8^	33	50
*Molinia caerulea*	-	-	-	-	8 ^26.5^	25 ^46.6^	-	-	-	-	-
*Parnassia palustris*	-	67 ^63.1^	-	7	17 ^1.4^	-	-	-	18 ^23.7^	17 ^20.8^	-
*Pedicularis palustris*	-	-	-	-	-	-	75 ^84.5^	-	-	-	-
*Peucedanum palustre*	17	33	67 ^3.6^	69 ^22.1^	100 ^34.4^	-	-	40 ^46.7^	-	17 ^11^	-
*Phragmites australis*	17 ^4.2^	17 ^4.2^	14^1^	-	33 ^26^	25 ^18.4^	-	-	45 ^46.8^	-	-
*Picea abies*	50 ^57.6^	-	5	-	8	25 ^31.8^	-	-	-	17 ^17.1^	-
*Pinguicula vulgaris*	-	17 ^17.7^	24 ^30.4^	-	-	-	-	-	-	-	-
*Pinus sylvestris*	-	-	-	-	8 ^26.5^	25 ^12.5^	25 ^12.5^	40 ^31.3^	-	-	-
*Poa palustris*	33 ^54.2^	-	-	-	-	-	75 ^58.6^	-	36 ^16.4^	17	-
*Poa trivialis*	-	-	-	-	-	50 ^20^	-	40 ^10.2^	27	-	60 ^26.9^
*Potentilla erecta*	-	33 ^20^	33 ^20^	7 ^8^	-	25 ^46.6^	-	-	-	-	-
*Ranunculus lingua*	50 ^59.6^	-	-	7	-	-	100 ^55.9^	80 ^37.6^	27	17	10
*Rhynchospora alba*	-	17	5	-	-	-	-	-	-	-	-
*Rumex acetosa*	67 ^34.5^	-	52 ^20.7^	-	67 ^34.5^	25	25	80	91	83	100
*Salix aurita*	17 ^9^	-	5	-	42 ^45.4^	25 ^37.3^	-	-	9 ^6.6^	-	-
*Salix rosmarinifolia*	33	33	57 ^11.1^	23	92 ^42.1^	50 ^32.6^	-	40 ^21.5^	-	33 ^14.1^	-
*Saxifraga hirculus*	-	17 ^10.9^	-	-	-	-	-	-	-	-	-
*Silene flos-cuculi*	33 ^54.2^	-	-	-	-	50 ^1.7^	-	80 ^28.6^	82 ^30.2^	67 ^16.6^	10
*Stellaria palustris*	67 ^63.9^	17 ^1.7^	-	-	8	-	-	-	64^21^	100 ^54.1^	80 ^35.9^
*Thelypteris palustris*	-	-	5	7 ^6.3^	17 ^22.9^	-	-	40 ^45.8^	18 ^12.8^	-	-
*Typha* sp.	-	-	5 ^20^	-	-	-	-	-	82 ^65.9^	17	30 ^9.4^
*Trichophorum alpinum*	17	67 ^16.1^	57 ^7.6^	23 ^8.2^	75 ^23.5^	-	-	20 ^41.5^	-	-	-
*Triglochin palustris*	-	83 ^69.7^	10	23 ^8.2^	-	25 ^2.5^	75 ^56^	-	9	17	10
*Utricularia intermedia*	-	-	-	23 ^6.8^	8	-	-	-	-	17	-
*Vaccinium oxycoccos*	50	100 ^19.7^	81	84 ^19.7^	92 ^9.6^	-	-	80 ^76.9^	18 ^2.2^	-	-
*Viola* sp.	33 ^20.7^	-	14	7 ^13.5^	17 ^0.5^	50 ^34.8^	-	40 ^23.5^	9	17	-

## Appendix B

**Table A2:** Results of TWINSPAN classification showing species presence, frequency, and *φ* coefficients for indicator species across different bryophyte groups (1B–10B). Columns represent bryophyte groups, rows represent species, and each cell contains species frequency with the *φ* coefficient indicated as a superscript. Dominant species were those with a frequency greater than 50% and a *φ* coefficient above 30 (*φ* coefficient expressed as a percentage, i.e., multiplied by 100), indicating frequent occurrence and strong association with the respective group.

Bryophyte Groups	1B	2B	3B	4B	5B	6B	7B	8B	9B	10B
No. of relevés	8	7	12	10	17	7	8	10	6	13
Species										
*Aneura pinguis*	-	-	8	33 ^48^	28 ^9.3^	-	-	-	-	-
*Aulacomnium palustre*	-	-	25 ^42.1^	-	-	50 ^28.3^	-	60 ^35.6^	100 ^54.6^	77 ^44.5^
*Brachythecium mildeanum*	-	-	-	-	-	-	-	20 ^34^	-	54 ^77.6^
*Calliergon giganteum*	38 ^24.2^	-	-	-	16	28	75 ^30.2^	-	16 ^9.3^	-
*Calliergonella cuspidata*	-	28	-	-	-	57 ^28.2^	100 ^42.2^	100 ^42.2^	83 ^31.8^	100 ^42.2^
*Campylium stellatum*	100 ^45.3^	100 ^45.3^	100 ^45.3^	100 ^45.3^	72 ^18.1^	57	-	-	-	-
*Cinclidium stygium*	71 ^64.5^	-	25 ^12.3^	55 ^32.4^	100 ^77.1^	-	12	-	-	-
*Climacium dendroides*	-	-				14 ^32.2^	-	10	-	7
*Drepanocladus aduncus*	-	-	-	-	-	-	-	50 ^64.4^	16 ^15.7^	30 ^30.2^
*Drepanocladus trifarius*	-	-	-	100 ^78.9^	-	-	-	-	-	-
*Hamatocaulis vernicosus*	-	-	-	-	-	28 ^15.6^	100 ^65.5^	90 ^60.2^	-	38 ^28.2^
*Helodium blandowii*	14 ^1.8^	-	-	-	-	-	-	-	33 ^24.4^	77 ^59^
*Marchantia polymorpha*	-	-	-	-	-	-	100 ^49.6^	80 ^42.2^	83 ^46.6^	84 ^49^
*Meesia triquetra*	-	-	-	20 ^19.2^	11 ^14.1^	-	-	-	-	-
*Paludella squarrosa*	14 ^1.8^	-	-	-	-	28 ^12.4^	-	10 ^1.3^	100 ^68.9^	23 ^10.3^
*Philonotis fontana*	-	-	-	-	5	-	-	-	-	-
*Plagiomnium elatum*	-	28 ^22^	58 ^62^	33 ^37^	-	-	-	-	16 ^11.3^	-
*Plagiomnium ellipticum*	-	-	-	-	-	71 ^33^	62 ^27.9^	100 ^48^	100 ^48^	92 ^43^
*Polytrichum strictum*	14 ^32.8^	-	-	-	-	-	-	-	-	7
*Ptychostomum pseudotriquetrum*	100	100	100	100	100	100	100 ^6.9^	100 ^6.9^	100 ^6.9^	84
*Scorpidium cossonii*	100 ^39.8^	100 ^39.8^	100 ^39.8^	100 ^39.8^	94 ^24.2^	57 ^12.3^	-	-	-	-
*Scorpidium scorpioides*	28 ^11.2^	-	66 ^32.2^	88 ^68.2^	88 ^68.2^	-	-	-	-	-
*Sphagnum contortum*	57 ^72.2^	-	-	-	11 ^14.1^	-	-	-	-	-
*Sphagnum divinum*	-	-	-	-	-	-	-	-	-	7
*Sphagnum teres*	-	-	91 ^86.2^	-	-	57 ^38.4^	-	10 ^6.3^	100 ^60.7^	15 ^8.9^
*Sphagnum warnstorfii*	-	-	-	-	-	28 ^48.2^	-	-	-	15 ^34.7^
*Tomentypnum nitens*	-	-	50 ^47.3^	-	-	14 ^1.8^	12 ^1.5^	100 ^53.9^	100 ^53.9^	100 ^53.9^
